# Acknowledgment of Reviewers

**DOI:** 10.2188/jea.JE20130146

**Published:** 2013-11-05

The following scientsts assisted the journal by reviewing manuscripts during the period October 1, 2012 to September 30, 2013. We gratefully acknowledge their critical evaluation and assistance in selecting articles for publication.

**Table:** 

Adiatman, Melissa	University of Indonesia
Aida, Jun	Tohoku University Graduate School of Dentistry
Ando, Ryosuke	Nagoya City University Graduate School of Medical Sciences
Ansai, Toshihiro	Kyushu Dental University
Arisawa, Kokichi	Institute of Health Biosciences, the University of Tokushima Graduate School
Asakura, Keiko	Keio University School of Medicine
Asao, Keiko	University of Michigan Health System
Asayama, Kei	University of Leuven
Azuma, Koichirou	Keio University
Babazono, Akira	Kyushu University
Breslow, Rosalind	National Institutes of Health
Chaves, Paulo	Florida International University
Cologne, John	Radiation Effects Research Foundation
Daisuke, Ekuni	Okayama University
Eguchi, Eri	Ehime University
Fujiwara, Takeo	National Institute of Public Health
Fukuda, Yoshiharu	Yamaguchi Unversity School of Medicine
Furuta, Michiko	Kyushu University
Hara, Megumi	Saga University
Hashizume, Masahiro	Institute of Tropical Medicine, Nagasaki University
Hata, Jun	Graduate School of Medical Sciences, Kyushu University
Hayasaka, Shinya	Hamamatsu University School of Medicine
Hayashi, Kunihiko	Gunma University
Hayashida, Kenshi	University of Occupational and Environmental Health
Higashiyama, Aya	Hyogo College of Medicine
Hirose, Kaoru	Aichi Prefectural Institute of Public Health
Hishida, Asahi	Nagoya University Graduate School of Medicine
Holick, Michael	Boston University Medical Center
Honda, Sumihisa	Nagasaki University Graduate School of Biomedical Sciences
Honda, Yasushi	University of Tsukuba
Honjo, Kaori	Osaka University Global Collaboration Center
Honjo, Satoshi	Fukuoka East Medical Centre
Hosoi, Toshiyuki	National Center for Geriatrics and Gerontology
Hozawa, Atsushi	Tohoku Medical Megabank Organization
Imaeda, Nahomi	Nagoya Women’s University
Inoue, Shigeru	Tokyo Medical University
Ishihara, Junko	Sagami Women’s University
Ishikawa, Hirofumi	Okayama University
Ishitake, Tatsuya	Kurume University School of Medicine
Ishizaki, Tatsuro	Tokyo Metropolitan Institute of Gerontology
Ito, Yuri	Osaka Medical Center for Cancer and Cardiovascular Disease
Iwai, Naoharu	National Cerebral and Cardiovascular Center Research Institute
Iwasa, Hajime	Fukushima Medical University School of Medicine
Jwa, Seung-chik	National Research Institute for Child Health and Development
Kaneko, Yoshihiro	Akita University School of Medicine
Katanoda, Kota	National Cancer Center
Kawaguchi, Yoko	Tokyo Medical and Dental University
Kawamura, Takashi	Kyoto University
Kikuchi, Shogo	Aichi Medciacl University School of Medicine
Kita, Yoshikuni	Shiga University of Medical Science
Kitano, Naomi	Wakayama Medical University School of Medicine
Kobashi, Gen	National Institute of Radiological Sciences
Kojima, Masayo	Nagoya City University
Kokubo, Yoshihiro	National Cardiovascular Center
Kondo, Naoki	University of Tokyo School of Public Health
Kotani, Kazuhiko	Jichi Medical University
Kubo, Tatsuhiko	University of Occupational and Environmental Health
Kuriki, Kiyonori	Laboratory of Public Health, School of Food and Nutritional Sciences
Kuriyama, Nagato	Kyoto Prefectural University of Medicine
Kurokawa, Naoyuki	Miyagi University of Education
Kurozawa, Youichi	Tottori University
Liao, Chung-Min	National Taiwan University
Lin, Yingsong	Aichi Medical Uniersity
Liu, Ching-Chuan	National Cheng Kung University
Ma, Enbo	University of Tsukuba
Mahbub, Hossain	Yamaguchi University School of Medicine
Matsuda, Tomohiro	Center for Cancer Control and Information Services, National Cancer Center
Matsumoto, Koji	University of Tsukuba
Matsumura, Yasuhiro	Bunkyo University
Matsushima, Masato	Jikei University School of Medicine
Matsushita, Yumi	National Center for Global Health and Medicine
Metoki, Hirohito	Tohoku University School of Medicine
Michikawa, Takehiro	National Institute for Environmental Studies
Minami, Yuko	Tohoku University School of Health Sciences
Miyachi, Motohiko	National Institute of Health & Nutrition
Miyake, Yoshihiro	Faculty of Medicine, Fukuoka University
Miyano, Ichiro	Kochi University
Miyatake, Nobuyuki	Kagawa University
Mizoue, Tetsuya	International Medical Center of Japan
Mori, Mitsuru	Sapporo Medical University
Mori, Rintaro	National Center for Child Health and Development
Murakami, Yoshitaka	Shiga University of Medical Science
Muraki, Shigeyuki	The University of Tokyo
Murase, Norio	Tokyo Medical University
Nagai, Masaki	Saitama Medical University
Nagano, Jun	Kyushu University
Nagata, Chisato	Gifu University Graduate School of Medicine
Naito, Mariko	Nagoya University Graduate School of Medicine
Nakamura, Kazutoshi	Niigata University
Nakamura, Koshi	Kanazawa Medical University
Nakamura, Yasuyuki	Kyoto Women’s University
Nakata, Yoshio	University of Tsukuba
Nakaya, Tomoki	Ritsumeikan University
Nakayama, Takeo	Kyoto University
Nishikitani, Mariko	Fukuoka Women’s University
Nishimura, Kunihiro	National Cardiovascular and Cerevral Center
Nishiwaki, Yuji	Toho University
Nishiyama, Takeshi	Nagoya City University
Nojima, Masanori	Sapporo Medical University, School of Medicine
Obara, Taku	Tohoku University
Obuchi, Shuichi	Tokyo Metropolitan Institute of Gerontology
Ohfuji, Satoko	Osaka City University Faculty of Medicine
Ohkado, Akihiro	Research Institute of Tuberculosis, Japan Anti-Tuberculosis Association
Ohnishi, Hirofumi	Sapporo Medical University School of Medicine
Ohsawa, Masaki	Iwate Medical University
Oida, Yukio	Chukyo University
Ojima, Toshiyuki	Hamamatsu University School of Medicine
Oka, Rie	Hokuriku Central Hospital
Okada, Katsutoshi	Ehime University
Okada, Rieko	Nagoya University Graduate School of Medicine
Okamoto, Etsuji	National Institute of Public Health
Okamoto, Kazushi	Aichi Prefectural College of Nursing and Health
Okamoto, Naoyuki	Kanagawa Cancer Center
Okayama, Masanobu	Jichi Medical University, Center for Community Medicine
Okumura, Junko	Nagasaki University, Institute of Tropical Medicine
Onozuka, Daisuke	Fukuoka Institute of Health and Environmental Sciences
Ooki, Syuichi	Ishikawa Prefectural Nursing University
Oshitani, Hitoshi	Tohoku University Graduate School of Medicine
Otsuka, Rei	National Center for Geriatrics and Gerontology
Ozasa, Kotaro	Radiation Effects Research Foundation
Oze, Isao	Aichi Cancer Center Research Institute
Pham, Ngoc Minh	National Center for Global Health and Medicine
Pham, Truong-Minh	Alberta Health Services-Cancer Care
Raoult, Didier	Aix Marseille Université
Sadakane, Atsuko	Radiation Effects Research Foundation
Saijo, Yasuaki	Asahikawa Medical College
Saika, Kumiko	Center for Cancer Control and Information Services, National Cancer Center
Sairenchi, Toshimi	Dokkyo Medical University School of Medicine
Saito, Isao	Ehime University Graduate School of Medicine
Sakamoto, Wataru	Okayama University
Sakuma, Naoko	Tokyo Metropolitan Institute of Gerontology
Sakurai, Masaru	Kanazawa Medical University
Sasazuki, Shizuka	National Cancer Center
Sato, Shinichi	Chiba Prefectural Institute of Public Health
Satoh, Toshihiko	Kitasato University
Sawada, Norie	Research Center for Cancer Prevention and Screening, National Cancer Center
Shibata, Akiko	Center for Cancer Control and Information Services National Cancer Center
Shima, Masayuki	Hyogo College of Medicine
Shimazu, Taichi	National Cancer Center
Shinozaki, Keiko	Yamaguchi University
Shobugawa, Yugo	Niigata University
Sone, Hirohito	University of Tsukuba Institute of Clinical Medicine
Sugiyama, Daisuke	Keio University
Sugiyama, Hiromi	Radiation Effects Research Foundation
Sumi, Ayako	Sapporo Medical University School of Medicine
Suzuki, Etsuji	Graduate School of Medicine, Dentistry and Pharmaceutical Sciences, Okayama University
Suzuki, Kohta	University of Yamanashi, Interdisciplinary Graduate School of Medicine and Engineering
Suzuki, Kunitake	Osaka University of Human Science
Tabara, Yasuharu	Kyoto University Graduate School of Medicine
Tabuchi, Megumi	Kwansei Gakuin University
Taguri, Masataka	Yokohama City University
Takahashi, Kunihiko	National Institute of Public Health
Takebayashi, Toru	Keio University
Takeshita, Tatsuya	Wakayama Medical University
Takeuchi, Fumihiko	National Institute of Infectious Diseases
Takezaki, Toshiro	Kagoshima University
Tamaki, Junko	Kinki University Faculty of Medicine
Tamakoshi, Koji	Nagoya University School of Health Sciences
Tamashiro, Hiko	Hokkaido University
Tanabe, Naohito	Niigata University Graduate School of Medical and Dental Sciences
Tanaka, Hideo	Aichi Cancer Center Research Institute
Tanaka, Junko	Hiroshima University Graduate School of Biomedical Sciences
Tanaka, Keitaro	Saga University
Tanaka, Shiro	Kyoto University
Tanihara, Shinnichi	Fukuoka University
Tokudome, Shinkan	National Institute of Health and Nutrition
Toyoshima, Hideaki	Anjo Kosei Hospital
Tsuji, Satoshi	National Center for Child Health and Development
Tsukuma, Hideaki	Osaka Medical Center for Cancer and Cardiovascular Disease
Turin, Tanvir	University of Calgary
Uemura, Kazuki	National Center for Geriatrics and Gerontology
Umesawa, Mitsumasa	Center for Medical Sciences
Wakai, Kenji	Nagoya University Graduate School of Medicine
Washio, Masakazu	St. Mary’s College
Watanabe, Makoto	National Cardiovascular Center
Watanabe, Shuichiro	J. F. Oberlin University
Yamagata, Zentaro	University of Yamanashi
Yamagishi, Kazumasa	University of Tsukuba
Yamaguchi, Naohito	Tokyo Women’s Medical University
Yamaji, Taiki	National Cancer Center
Yamamoto, Seiichiro	National Cancer Center
Yamazaki, Shin	Kyoto University School of Public Health
Yano, Eiji	Teikyo University
Yatsuya, Hiroshi	Fujita Health University
Yokokawa, Hirohide	Fukushima Medical University School of Medicine
Yokomichi, Hiroshi	University of Yamanashi
Yonemoto, Naohiro	National Center of Neurology and Psychiatry
Yoshihara, Akihiro	Graduate School of Medical and Dental Science, Niigata University

